# Water in Crystalline Fibers of Dihydrate β-Chitin Results in Unexpected Absence of Intramolecular Hydrogen Bonding

**DOI:** 10.1371/journal.pone.0039376

**Published:** 2012-06-19

**Authors:** Daisuke Sawada, Yoshiharu Nishiyama, Paul Langan, V. Trevor Forsyth, Satoshi Kimura, Masahisa Wada

**Affiliations:** 1 Department of Biomaterials Sciences, Graduate School of Agricultural and Life Sciences, The University of Tokyo, Tokyo, Japan; 2 Centre de Recherches sur les Macromolécules Végétales (CERMAV-CNRS), BP 53, F-38041Grenoble, France; 3 Biology and Soft Matter Division, Oak Ridge National Laboratory, Oak Ridge, Tennessee, United States of America; 4 Partnership for Structural Biology, Institut Laue Langevin, Grenoble, France; 5 EPSAM/ISTM, Keele University, Keele, Staffordshire, United Kingdom; 6 Department of Plant & Environmental New Resources, College of Life Sciences, Kyung Hee University, Seocheon-dong, Giheung-ku, Yongin-si, Gyeonggi-do, Korea; Consejo Superior de Investigaciones Cientificas, Spain

## Abstract

The complete crystal structure (including hydrogen) of dihydrate β-chitin, a homopolymer of N-acetylglucosamine hydrate, was determined using high-resolution X-ray and neutron fiber diffraction data collected from bathophilous tubeworm *Lamellibrachia satsuma*. Two water molecules per N-acetylglucosamine residue are clearly localized in the structure and these participate in most of the hydrogen bonds. The conformation of the labile acetamide groups and hydroxymethyl groups are similar to those found in anhydrous β-chitin, but more relaxed. Unexpectedly, the intrachain O3-H…O5 hydrogen bond typically observed for crystalline β,1–4 glycans is absent, providing important insights into its relative importance and its relationship to solvation.

## Introduction

Linear extended polysaccharides such as cellulose and chitin form crystalline fibrous materials that play important mechanical roles in biological systems. They also represent the major part of biomass that can be transformed into high-performance industrial materials, fuels, basic chemicals and other biocompatible products. Understanding the structural and chemical properties of these feedstock materials can be of fundamental importance to the development of more efficient or new processing conditions. Thus recent determination of the complete structures of the various crystal forms of cellulose using high resolution fiber diffraction[1]–[11] has helped guide new approaches to the efficient conversion of cellulosic biomass into biofuels. [12], [13] In these studies X-rays were used to visualize the arrangement of carbon and oxygen atoms that make up the molecular skeleton of the cellulose chains, and neutrons were used to visualize the smaller and more mobile hydrogen atoms involved in hydrogen bonding. In this work we applied this complementary X-ray and neutron fiber diffraction approach to the study of chitin and obtained the complete structure and hydrogen-bond arrangement for one of its more biologically and industrially important crystal forms, namely β-chitin dihydrate.

Chitin, composed of β-(1–4)-linked N-acetyl-D-glucosamine, is the second most abundant structural biopolymer on earth next to cellulose [14], [15]. Its molecular structure is similar to that of cellulose, except that hydroxyl is replaced by an acetamide at the C2 position. Two major naturally occurring crystal allomorphs, namely α-chitin and β-chitin, have been identified from their distinct X-ray diffraction patterns, infrared spectra and NMR spectra[14]–[16]. The α-chitin allomorph is found mainly in arthropod cuticles whereas β-chitin is found in the extracellular filaments of diatoms, the tensile reinforcement elements in squid tendon, pogonophores and vestimentiferans tubes [15], [17], [18]. Only α-chitin has been obtained when the polymer is recrystallized from solution.

The crystal structure of anhydrous β-chitin [19], recently determined at atomic resolution using X-ray fiber diffraction (*a* = 4.819 Å; *b* = 9.239 Å; *c* = 10.384 Å; *γ* = 97.16°; space group *P*2_1_), corresponds to a one-chain unit cell with one N-acetyl-D-glucosamine residue in the asymmetric unit and a *gg* conformation for the hydroxymethyl group. The structure is stabilized in all directions by van der Waals interactions and hydrophobic effects, but hydrogen bonding is absent in the direction of the *b*-axis. β-chitin can incorporate various small molecules, such as water, alcohols, and amines[20]–[22] into its crystal lattice to form crystalline complexes (crystallosolvates). The most important and readily formed complexes are the hydrates. In early X-ray fiber diffraction studies Blackwell and coworkers reported that β-chitin could be found in several different structures depending on the exact conditions of sample preparation [23]. For purified β-chitin, two distinct hydrate structures were identified, both with the same fiber repeat of 10.4 Å, monoclinic angle of 97°, *a* = 4.8 Å but differing in the length of the last axis (the *b*-axis in today’s convention). The crystal structure of anhydrous β-chitin therefore appears to swell in the *b*-axis direction in order to accommodate the water molecules. Molecular model building studies indicated that the two structures corresponded to mono- and dihydrates, although no complete structural model of dihydrate β-chitin has been reported, possibly due to the limited resolution of the X-ray data and the relatively large number of degrees of freedom in classical linked atom least squares refinement approaches.

More recently, Kobayashi and coworkers studied the reversible hydration and dehydration behavior of β-chitin and several other polysaccharides by changing the relative humidity of the sample environment[24]–[27]. β-chitin showed abrupt and stepwise transitions between different hydration states [24] in contrast to the more continuous transitions observed in the dehydration of water-cellulose [25], [26] or the hydration and de-hydration of paramylon [27]. The hydration and dehydration of β-chitin by humidity control also shows a strong hysteresis: dihydrate β-chitin is formed by direct contact with water and is stable in relative humidities above 30% at ambient temperature. The mono-hydrate has a very narrow window of existence and is obtained by raising the relative humidity of anhydrous β-chitin to above 80% or by drying dihydrate β-chitin at 30% R.H.

In this study, we have determined the complete (including hydrogen atoms) atomic resolution crystal structure of dihydrate β-chitin. One of the hydrogen positions, and thus the hydrogen-bond scheme, obtained by neutron diffraction data was different from its predicted position based on the geometry of oxygen, carbon and nitrogen atom in the X-ray structure, revising our perception of 1,4 β glucan chains. This study demonstrates the power of combining X-ray and neutron diffraction techniques with advances in sample preparation methods for providing atomic level information on the structure and properties of industrially important biomasses.

## Results and Discussion

**Figure 1:**
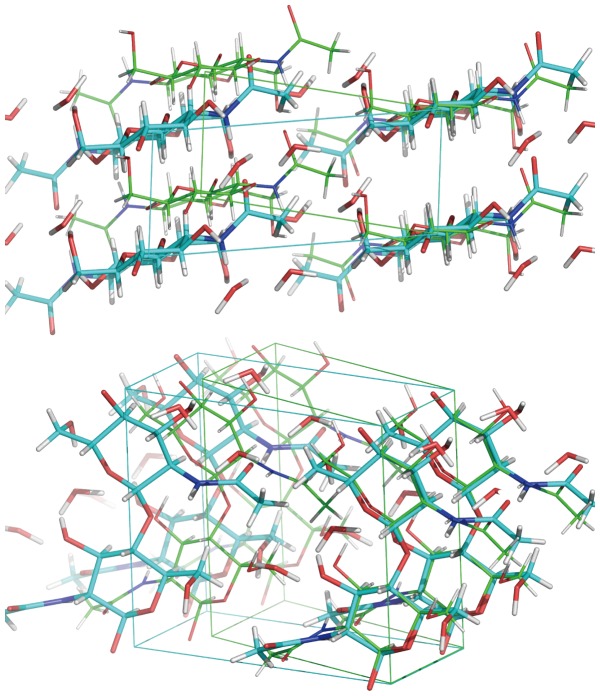
Superposition of dihydrate β-chitin (cyan backbone and cell) and anhydrous structure (green).

The crystallographic unit cell parameters for dihydrate β-chitin determined in this study, *a* = 4.814 Å *b* = 11.167 Å *c* = 10.423 Å *γ* = 96.45°, are similar to those previously reported (*a* = 4.8 Å *b* = 11.1 Å *c* = 10.4 Å *γ* = 97°) [23] or (*a* = 4.80 Å *b* = 11.15 Å *c* = 10.44 Å *γ* = 96.39°) [24]. There are two water molecules per N-acetyl-D-glucosamine residue, confirming the hydration number determined by gravimetric analysis [24]. The interconversion of β-chitin between its dihydrate and anhydrous crystal forms is accompanied by a small change in the spacing between stacked sheets (which interact through hydrogen bonds between acetamide groups and hydrophobic interactions), and a larger shearing of the sheets by about 10 or 14 degrees depending on the shear directions (Figure 1).

The unit cell volume of dihydrate β-chitin (containing two GlcNac residues and 4 water molecules) is 556.8 Å^3^ compared to 459.4 Å^3^ for anhydrous β-chitin (containing just two GlcNac residues) [19]. If we consider the molecular volumes associated with the density of bulk water and anhydrous β-chitin, then we might expect a unit cell volume for a binary complex containing two GlcNac and 4 water molecules of 579 Å^3^, i.e. about 4% larger than the experimental value. The more compact structure is an indication of the enthalpic advantage of hydration due to London dispersion forces.

**Figure 2:**
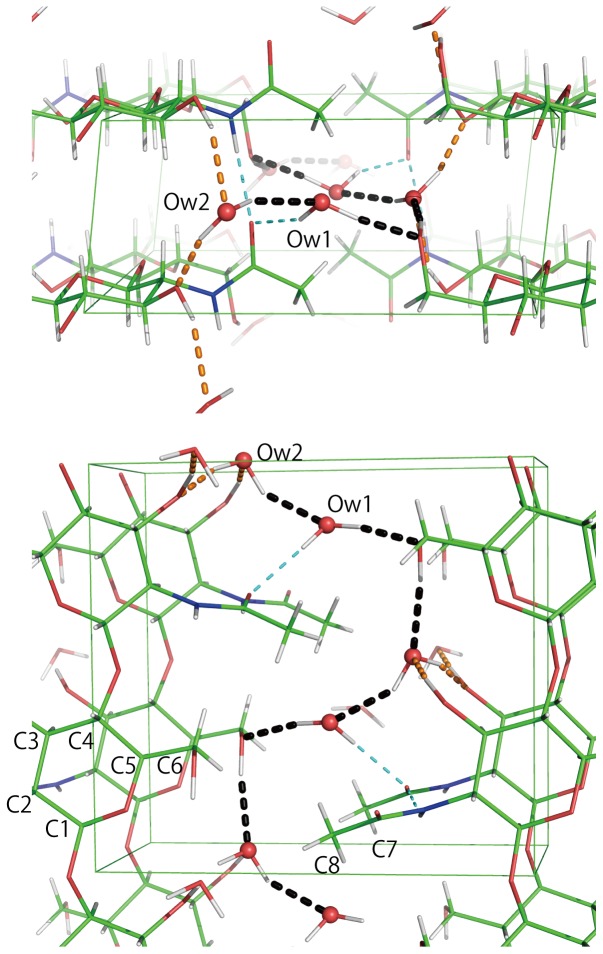
Final structure model and hydrogen bond networks of dihydrate β-chitin. It is seen along the chain direction and ab projection.

The hydrogen-bond geometry of the final structure (model A) is summarized in Table 1(Hydrogen bonds are listed using the criteria: distance between donor and acceptor is shorter than the sum of their van der waals radii +0.5 Å; distance deuterium atom to acceptor is shorter than sum of corresponding van der waals radii - 0.12 Å; Donor-D…Acceptor angle is greater than 100 degree.) and is shown in Figure 2. Both anhydrous β-chitin [28] and dihydrate β-chitin have an intersheet hydrogen bond between acetamide groups in stacked sheets (N2-D…O7) that acts as a hydrogen-bond bridge between the sheets. The primary alcohol to acetamide oxygen O6-H…O7 hydrogen bond that exists in anhydrous β-chitin does not occur in dihydrate β-chitin. This is not surprising as the hydrogen-bond geometry in the anhydrous structure is far from ideal. However, the absence of the intrachain O3-H…O5 hydrogen bond in dihydrate β-chitin is surprising. Rather, the deuterium atom on O3 is donated in a hydrogen bond to a water molecule.

**Table 1:** Hydrogen bonding geometry.

Do	A	D…A distance(Å)	Do-D…A angle(°)	D…A-D’ angle(°)	A residue
N2	O7	1.96(10)	150 (7)	–	x+1,y,z
O3	Ow2	2.21(17)	153 (9)	70(Ow1), 120(O3)	x,y,z
O6	Ow2	2.00 (13)	176 (11)	137(Ow1), 117(O3)	−x,−y,−1/2+z
Ow1	O7	1.93 (14)	159 (14)	–	x+1,y,z
Ow1	O6	1.79 (11)	167 (15)	102(Ow2)	x+1,y+1,z
Ow2	O3	1.68 (16)	166 (15)	96(Ow2)	x+1,y,z
Ow2	Ow1	1.96 (15)	156 (13)	144(O6), 76(7)	x,y,z
C2	O7	2.39	103	–	x,y,z
C6	O1	2.51	102	–	−x,−y,1/2+z
C8	Ow1	2.53	154	99(O6), 83(O7)	x,y,z

D: deuterium Do: donor A: acceptor.

This result is unexpected because we have found the O3-H…O5 intrachain hydrogen bond to be present in the crystal structures of anhydrous β-chitin and in all the various allomorphs of cellulose that we have studied so far using X-ray and neutron fiber diffraction. It is thought to confer mechanical and conformational stiffness to β,1–4 glycan chains and its formation is related to the conformational parameters τ and ψ that describe the glycosidic linkage. In crystals of cellulosic small molecules French and Johnson have always observed its presence when ψ <−130°, if a hydroxyl group is present at the O3 position. [29] When ψ is larger, the O3 and O5 atoms on neighboring residues are rotated away from each other so that they can no longer hydrogen bond. Two crystal structures of chitobiose have been reported by Mo and Jensen [30], and by Mo [31]. In β-(1–4) linked β-N,N’-diacetylchitobiose trihydrate (BCHITT10), the O3-H…O5 hydrogen bond is present, although it is bifurcated with a minor O3-H…O6 hydrogen bond component and the geometry is relatively distorted. In the β-(1–4) linked α-N,N’-diacetylchitobiose (ACHITM10) monohydrate structure the O3-H…O5 hydrogen bond is absent because the value of ψ is −106°. In the dihydrate β-chitin structure reported here, the values of τ and ψ are −147° and 115°, Table 2. These values are within the ranges observed for anhydrous β-chitin and the allomorphs of cellulose and should therefore allow the formation of the O3-H…O5 hydrogen bond. The absence of the O3-H…O5 hydrogen bond in dihydrate β-chitin cannot be explained by an unfavorable conformation of the glycosidic linkage.

**Table 2:** Conformational parameters of anhydrous β-chitin and dihydrate from the X-ray model.

	τ	ω	ω'	ϕ	ψ
This structure	115(2)	−54(3)	67(3)	−95(2)	−147(2)
Anhydrous form	117	−65	58	−89	−152

τ: glycosidic bond angle, torsion angles ω: O5C5C6O6, ω’: C4C5C6O6, ϕ: O5C1O1C4’, ψ: C1O1C4’C5’.

The conformations around glycosidic bonds have been also extensively studied from theoretical approaches including primitive molecular mechanics to quantum chemical calculations and molecular dynamics simulation. Despite the intuitive importance of hydrogen bonding, experimental results have been often better reproduced by artificially weakening the hydrogen bond effect by increasing the dielectric constant of the environment [32] or quantum chemical calculation of analogue molecules replacing the hydroxyl groups by Fluor atom [33]. Thus the intramolecular hydrogen bonding is not a prerequisite for the set of glycosidic angles in cellulose and chitin allomorphs giving a two fold screw symmetry with 10 Å per rise.

The main difference between the dihydrate β-chitin structure and those of anhydrous β-chitin and the cellulose allomorphs that we have previously studied would appear to be the presence of water molecules near the O3 hydroxyl group. It may be that the higher degrees of freedom of the complexed water molecules allow for the formation of a stronger hydrogen bond between water and O3 than between O3 and O5. In addition, statistical analyses of hydrate structures indicate that water molecules are stronger acceptors than hydroxyls or ether oxygens. [34] If O3 does preferentially donate hydrogen bonds to solvent molecules rather than O5, then we can make several observations. First, this would partly explain the preference observed in molecular dynamics simulations of O3 of cellulose oligomers in aqueous solution to donate hydrogen bonds to water rather than O5 [35]. It would also suggest that at the hydrated surfaces of crystalline fibers of chitin and cellulose the O3 hydroxyl groups will preferentially hydrogen bond with water rather than O5, and this is indeed what is observed in molecular dynamics simulations of cellulose [13]. However, it would also suggest that the rupture of the O3-H…O5 hydrogen bond is not as important for the dissolution of cellulose in alkali solutions as previously proposed [36].

Another implication is that it might be expected that in the binary complexes of cellulose or chitin with other mobile solvent molecules, that the O3 hydroxyl group will hydrogen bond with the solvent molecules rather than O5. In fact in the complex of ammonia with cellulose the hydrogen atom on O3 is found to be disordered, being donated in a hydrogen bond most of the time to O5 on the same chain, but rotating away to also be donating to the ammonia molecule part of the time [11].

Although the absence of the O3-H…O5 hydrogen bond does appear to be consistent with the preference of O3 to donate hydrogen in hydrogen bonds to more mobile solvent molecules when they are available, there is another possible contributing factor. We note that the O3-D…Ow2 hydrogen bonds participate in long homodromic chains of hydrogen bonds (O3→Ow2→O3’→) along the *a*-axis that might run along the entire length of a crystal domain. Another chain of hydrogen bonds (O6→Ow2→Ow1→O6’→) can be observed in *bc* plane. Jeffrey and Saenger, reporting on the hydrogen-bond strength in a number of carbohydrate crystals, [34] note that when chains of hydrogen bonds are compared to isolated hydrogen bonds, the bonds in chains tend to be stronger because of cooperative effects. We have previously observed the importance of cooperative effects in hydrogen bonding for stabilizing the cellulose III_I_ allomorph of cellulose [5], [37].

The torsion angle C4 C5 C6 O6 (ω’) of 67° corresponds to the hydroxymethyl group adopting the *gg* conformation, as in anhydrous chitin, Table 2. This conformation is the most common for glucopyranosides. The deviation of 7° from an ideal dihedral angle is probably due to the effects of hydrogen bonding with Ow2, which exposes a lone pair to DO6. A recent NMR study of α and β glucose in aqueous solution indicates that *gt* is the major conformation of the hydroxymethyl group [38]. This agrees with the occurrence of *gt* in the majority of glucose-based crystals. The observation of the *gg* rather than the *gt* conformation for the hydroxymethyl group of N-acetyl glucosamine may be due to the distant presence of the acetamide group or it may be due to crystal hydrogen bonding interactions. The acetamide group is rotated by about 30° around the C2–N2 bond with respect to its orientation in the anhydrous chitin structure. This is can be correlated with the establishment of a hydrogen bond between Ow1 and O7 and the rupture of the hydrogen bond between O6 and O7 that existed in the anhydrous structure.

From table 1 it can be seen that most of the hydrogen bonds in dihydrate chitin directly involve water molecules. The waters w1 and w2 form three moderate hydrogen bonds and one rather weak hydrogen bond each. O6 (-x, -y, 0.5+z), Ow2, O3 and Ow1 lie in a plane where O6 (-x, -y, 0.5+z) donates to Ow2 and Ow2 donates to Ow1 and O3. O3 (1+x, y, z) donates to Ow2 instead of donating to the ring oxygen O5 as mentioned above. Ow2, Ow1, O7, and O6 (1+x, 1+y, z) also lie in a plane with water oxygen Ow1 at the center, where Ow2 donates hydrogen bond to Ow1 and Ow1 donates to O7 and O6. In addition, Ow1 accepts from C8 methyl group and Ow2 from O3 a relatively weak hydrogen bond. Most of the hydrogen-acceptor distances fall between 1.89 and 2.16 Å, longer compared to the hydrogen-bond geometry found in anhydrous β-chitin or small molecule analogues. However, these distances agree well with the average hydrogen bonding distances for water molecules in hydrate crystals [39]. The NH…O = C hydrogen bond in dihydrate has a longer H…O distance and the NH…O alignment is more bent compared to that in anhydrous β-chitin. The O3-H…O5 intramolecular hydrogen bond in anhydrous β-chitin has a H…O distance of 1.76 Å and an angle of 174° compared to 2.17 Å and 150° for the O3-H…Ow2 hydrogen bond in dihydrate β-chitin. Apparently, the increased number of hydrogen bonds and the denser packing compensates for the slightly unfavorable individual hydrogen geometries in dihydrate β-chitin compared to those in the anhydrate chitin structure.

## Materials and Methods

### X-ray Experimental Section

#### Sample preparation

Satsuma tubeworms (*Lamellibrachia satsuma*) were collected from the sea off Kagoshima Bay at a depth of about 100 meter using a remotely operated vehicle, Hyper-Dolphin (JAMSTEC, Japan). The samples were purified and oriented as described previously [24]. In the tubeworm specimen, fibers tend to align in layers, creating significant bi-axial orientation. In preparing a bundle of such fibers for diffraction analysis, individual fibers were rotated randomly in the *ab* plane to create a sample with essentially uniaxial orientation of the crystallites parallel to the major axis of the fiber bundle, i.e. crystallographic c. This was done to minimize complications in the data- reduction process. The native hydrate sample was kept in a desiccator at 100% R.H. until just before the X-ray measurement.

#### Data collection

Synchrotron X-ray fiber diffraction data were collected at the beam line BL38B1 at SPring-8 (Hyogo, Japan). The oriented fiber was mounted perpendicular to the beam (λ = 1.0 Å) on a small brass sample holder on a goniometer head in a 100% R.H. air flow generated by a humidity generator (Shinyei SRG-1R). A fiber diffraction pattern was recorded using a flat imaging plate (IP) (R-Axis V, Rigaku) with an exposer time of 180 s. The sample-to-IP distance, about 170 mm, was calibrated using Si powder (d = 0.31355 nm) [40].

In the x-ray fiber diffraction pattern, Figure S1A, there are noticeable diffraction peaks in the meridian direction corresponding to (0 0 1) and (0 0 3) reflections, but their relative intensities are small (∼1% of the intensity of the (0 0 2) reflection). Therefore the space group symmetry was approximated as *P*2_1_. D-spacings of 37 reflections were measured using R-axis software, indexed with a monoclinic unit cell, and then used for least squares refinement of the unit cell parameters (d-spacing values are listed in table S1); *a* = 4.814(2) Å *b* = 11.167(8) Å *c* = 10.423(10) Å and *γ* = 96.45(1)°.

A polarization correction (with a linear coefficient of 0.83), background subtraction, and peak fitting were carried out as described previously [19]. The diffraction pattern was transformed to a polar-coordinate intensity matrix of 360 by 2000 elements, corresponding to 1° increment in azimuthal angle and a 100 micron interval in reciprocal polar radius. The background was evaluated for each radial trace using a bicubic spline function with a grid point every 50 pixels (Fig. S1C). The fitted intensity of each Miller index (individual reflection) was kept in a “crude intensity list” for calculation of Fourier syntheses. However for structure refinement we grouped neighboring reflections on the same layer line that were close enough to overlap (because of cylindrical averaging) into composite intensities in a “regrouped intensity list”.

#### Structure solution

X-ray structure refinement was carried out using previously described strategies for applying SHELX-97 [41] to fiber diffraction data [2]. The backbone of the recently reported atomic resolution structure of anhydrous β-chitin [19] was taken as the phasing model. Bond lengths and bond angles were restrained to the average values of the β-(1–4)-N-acetyl-D-glucosamine dimer trihydrate [31] using DFIX and DANG options in SHELX-97 with default standard deviations. An initial refinement was carried out with the hydroxymethyl group O6 and acetamide group C7 C8 O7 atoms removed and using the crude intensity list up to 1.2 Å resolution. The meridional intensities were also omitted. Both positional parameters and individual isotropic atomic displacement parameters were refined. This involved 163 amplitudes with Fo>4σ(Fo), 32 parameters, with 28 restraints. Two alternate structures corresponding to the chains pointing either up or down in the unit cell were refined, as defined French and Howley [42].

**Figure 3:**
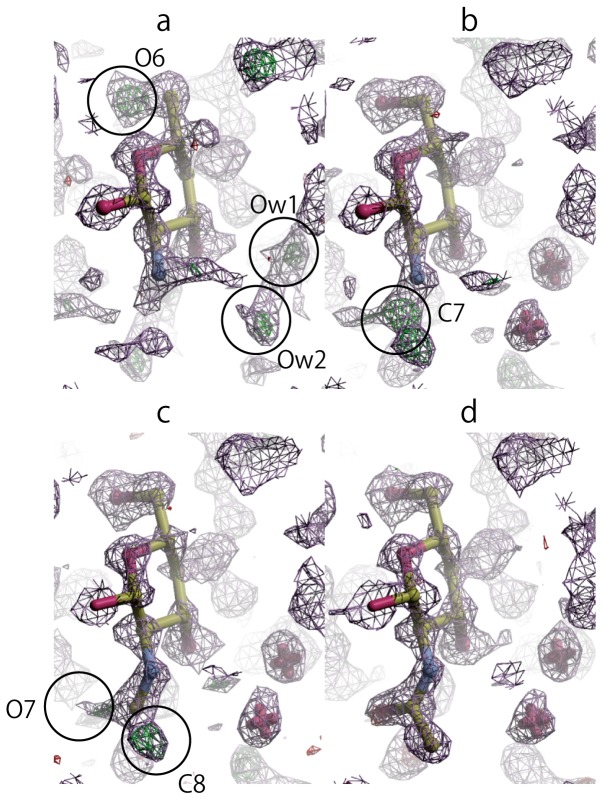
X-ray Fourier omit maps. Section through the X-ray σ_A_ map (showing positive density in blue) and the X-ray Fo-Fc omit map (showing positive and negative density in green and red, respectively). (a) Calculated using only the rigid backbone (omitting O6, C7, C8 and O7) as phasing model. Density indicated by circles can be associated with the hydroxymethyl oxygen O6 and two water molecules Ow1 and Ow2. (b) Calculated after introduction of O6 and the water oxygen atoms. Density indicated by circles can be associated with acetamide C7. (c) Calculated after further addition of C7. Density indicated by circles can be associated with C8, and O7. (d) Calculated with the complete molecular model (excluding hydrogen).

Both Fo-Fc and 2mFo-Fc (σ_A_) omit maps were calculated and then visualized by using *Coot* [43] and *Raster3D* [44] software. For the “down” model there were clear density peaks that could be associated with possible positions for the hydroxymethyl group O6 and the two water molecule oxygen atoms in the Fo-Fc map, Figure 3a. There were also peaks in the σ_A_ map that could be associated with the acetamide group C7 C8 O7 atoms. Refinement of the “down” model resulted in a value of 0.4272 for R1 (R1 is calculated from ∑(|Fo| - |Fc|)/∑|Fo| with Fo>4σ, where Fo and Fc are the observed and calculated amplitudes, respectively). Refinement of the “up” model resulted in a value of 0.5279 for R1. Thus the “up” model was readily discarded.

The missing atoms were then sequentially introduced at the positions of residual electron density peaks. Subsequent refinements were carried out with bond angle and bond distance restraints applied on the additional atoms whenever they were connected to the backbone. The sequential addition of O6, Ow1 and Ow2 (Fig. 3b), C7 (Fig. 3c), and finally C8 and O7 (trans to amine) (Fig. 3d), resulted in values of 0.4159, 0.3359, 0.3097, and 0.2647 for R1.

Further refinement was carried out using the regrouped intensity list, resulting in a reduction in the number of data (Fo>4σ(Fo)) to 97, and a value of 0.1153 for R1. Incorporation of hydrogen atoms attached to carbon and nitrogen atoms at standard positions using HFIX options resulted in a value of 0.1102 for R1. A refinement with isotropic atomic displacement parameters restrained with the SIMU option gave a value 0.0983 for R1. Finally, a refinement carried out with anisotropic atomic displacement parameters for non-hydrogen atoms, restrained with SIMU and DELU options, with the hydrogen atoms of hydroxyl groups added with HFIX options, resulted in a value 0.0781 for R1.

### Neutron Experimental Section

#### Sample preparation

Samples were purified and oriented as for the X-ray experiment and then inserted into soda-glass thin-walled capillaries. The loaded capillaries were then immersed in heavy water (D2O) and autoclaved at 160°C. The capillaries were then quickly sealed by flame and bundled to form arrays of 1 cm x 1 cm x 4 mm in size, large enough to fully exploit the relatively weak flux of the neutron beam. In this sample we expected all labile hydrogen atoms (attached to O and N) to be replaced by deuterium.

#### Data collection

Neutron diffraction data were collected using the four-circle D19 diffractometer at the Institut Laue-Langevin. The diffractometer is equipped with a large multiwire gas detector (30×120° angular aperture), a versatile monochromator and takeoff-angle assembly, and flexible arrangements for a variety of sample environment options [7]. The D19 detector has cylindrical geometry, and outputs a data array of size 256(vertically) x 640(horizontally), giving an angular resolution of 0.19° in the equatorial direction. Generic strategies for collecting fiber diffraction data on D19 have been described previously [45], [46], and the instrument has been used for the study of a wide range of synthetic and biological polymer systems[47]–[54]. A data set was collected at ambient conditions, using a neutron beam of wavelength 1.4558 Å, using several different sample goniometer settings, and over a measuring time of about 24 hours.

For each sample setting, the recorded data were corrected for spatial distortion using the D19 in-house program DCD19. The variation in response over the area of the detector was corrected for by dividing each data frame by a data frame collected from an isotropic incoherent scattering from vanadium rod. An affective absorption correction was determined by fitting a correction function that re-established a smooth incoherent scattering background. The neutron intensity data were extracted in a similar manner to the X-ray data, as described previously, and crude and regrouped intensity lists were generated.

#### Structure solution

**Figure 4:**
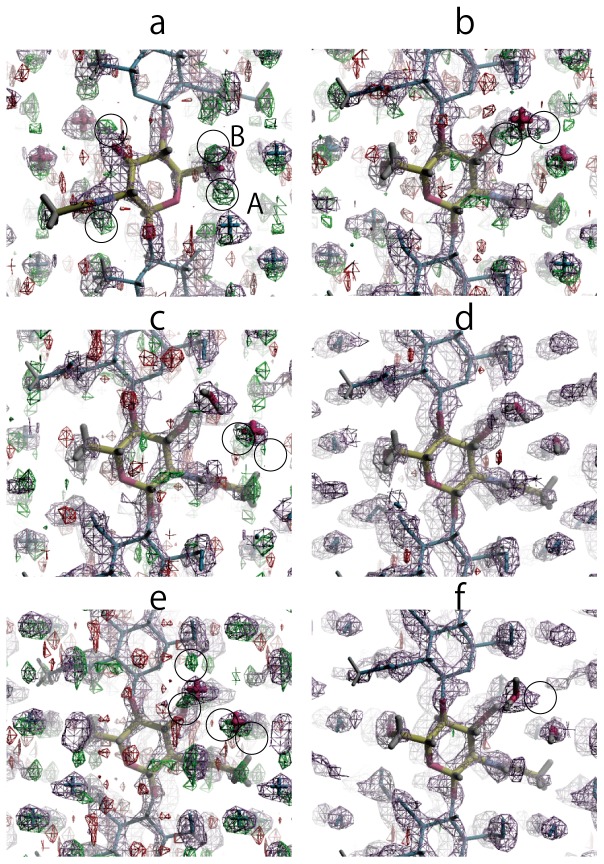
Neutron Fourier omit maps. Section through the neutron σ_A_ map (showing positive density in blue) and the Fo-Fc omit map (showing positive and negative density in green and red, respectively) after (a) removing all deuterium atoms (b) adding DO3, DN2 and DO6 at position A (c) further adding the deuterium atoms attached to Ow2 (d) further adding the deuterium atoms attached to Ow1 (e) adding DO3, DN2 and DO6 at position B (f) further adding the deuterium atoms attached Ow1 and Ow2. In (f) small residual peaks can still be seen near Ow2.

The backbone of the dihydrate β-chitin X-ray structure was taken as the phasing model. All hydrogen atoms (which we expect to have been substituted by deuterium) attached to hydroxyl groups and nitrogen atoms were removed from the model and only the overall scale factor was refined with the atomic positions and thermal displacement parameters kept fixed. At first only data up to 1.5 Å resolution were used for refinement (100 initial reflections with I>4σ and 84 reflections after removal of those with intensity 0 and with factor Fc/Fcmax < than 0.133), resulting in a value of 0.3446 for R1 after refinement. The resulting σ_A_ and Fo – Fc maps showed clear density peaks that could be associated with deuterium atoms attached to N2, O3, Ow1 and O6, Figure 4. For the deuterium atom attached to O6 there were two potentially associated peaks, which we label A and B, Figure 4a.

Deuterium atoms DN2 and DO3 were attached to N2 and O3, respectively, as indicated in the maps. There were two possible locations, A and B, for the deuterium atom DO6 attached to O6. A refinement with DO6 at position A, with O-D bond lengths for hydroxyl group restrained to 0.98 Å, N-D bond lengths restrained to 1.00 Å, and the C-O-D angles restrained to 110°, resulted in a value of 0.3144 for R1. The peak position corresponding to DN2 was slightly removed from acetamide plane but this plane was restrained to be flat at this stage. In the subsequent Fourier omit maps, density peaks could be clearly seen for the two deuterium atoms of one water molecule, Ow2, and for one of the deuterium atoms of the other water molecule, Ow1, Figure 4b. Introducing the two deuterium atoms attached to Ow2 to the refinement resulted in a value of 0.3024 for R1. O-D bond lengths for the water molecules were restrained to 0.97 Å and D-O-D angles were restrained to 106° [55]. In the subsequent Fourier omit maps density peaks appeared near Ow1 that could be associated with both of the attached deuterium atoms, Figure 4c. Adding the two deuterium atoms on Ow1 to the refinement resulted in a value of 0.2713 for R1, Figure 4d. This refined model was called model A.

In an alternative refinement, the DO6 atom was placed at location B and the same refinement process was followed. Refinement with deuterium atoms attached to O6, N2, and O3, and then with the deuterium atoms attached to Ow1 and Ow2 resulted in values of 0.3188 and 0.2754 for R1, respectively, Figure 4e. This refined model was called model B1. When the difference maps were calculated with all deuterium atoms in place, there was an additional density peak near Ow2 directed towards Ow1, and suggesting two additional alternative arrangements for the water deuterium atoms, which we call B2, and B3, and which resulted in values of 0.2730 and 0.2723 for R1. These four models, A, B1, B2 and B3, were further refined with the “regrouped intensity list” and data up to 1.50 Å resolution (85 reflections with I>4σ). The R1 values after refinement were 0.2899, 0.2855, 0.2913 and 0.2863 for model A and B1–B3, respectively. When the FLAT restraint on the acetamide group was lifted, the values of R1 increased to over 0.3 for all models and subsequent refinements were therefore we retained this FLAT restraint.

Including all reflections up to 1.32 Å in the refinement increased the number of intensities to 103 and resulted in values of 0.3023, 0.2998, 0.3038, 0.3037 for model A, B1, B2 and B3. Adding reflections beyond this resolution resulted in a dramatic increase in the value of R1 for all models, Figure S2A. Intensities with Fc/Fcmax <0.15 were omitted as to establish a flat analysis of variance, as described previously [2], Figure S2B, reducing the number of reflections to 94 and the values of R1 to 0.2621, 0.2654, 0.2658 and 0.2617 for model A, B1, B2 and B3, respectively. The 4 models were then further refined with individual isotropic thermal displacement parameters with SIMU restrains, resulting values of 0.2591, 0.2578, 0.2641 and 0.2604 for R1, for A, B1–B3, respectively. These values of R1 are higher than the values typically obtained with refinements of cellulose using neutron data at similar resolutions. Allowing all the atomic coordinates to refine together with a single parameter which scaled the individual atomic displacement parameters obtained from the X-ray structure resulted in values of 0.2229, 0.2191, 0.2419 and 0.2314, for R1, for A, B1–B3, with only slight changes in the positions of atoms.

Model B1 could be rejected because of an over-short close contact between a deuterium attached to Ow2 and O6. Model B2 and model B3 had significantly higher values of R1 than model A, and also had fewer hydrogen bonds than model A, with one of the deuterium atoms attached to Ow1 apparently participating in no hydrogen-bond interactions. On the other hand all of the deuterium atoms in model A participated in hydrogen-bond interactions.

Distance restraints were applied between donor atom Ow1 and its acceptor atom and O6 (1+x, 1+y, z) and donor atom Ow2 and its acceptor atom Ow1 to straighten the geometry of the corresponding hydrogen bonds. This did not significantly increase the value of R1.This geometrically idealized model A was taken as the final structure of dihydrate β-chitin.

The coordinates of the X-ran and neutron final structure are available as a Crystallographic Information File (cif format) in the file Dataset S1.

## Supporting Information

Figure S1 **Diffraction data of dihydrate β-chitin X-ray (A) and neutron (B) fiber diffraction data.** The fiber direction (meridian) is vertical and the equator is horizontal. Background scattering was subtracted using a rolling-ball algorithm for the purpose of improved visual presentation. X-ray fiber diffraction diagrams transformed into Polar coordinates. (C): observed (D): fitted. The origin of azimuthal angle corresponds to horizontal left of fiber pattern in (A).
(JPG)

Figure S2 **Data selection from R1 and K.** (A) R1 values calculated for reflections grouped in resolution shell. (B) K, the mean of Fo^2^ over the mean of Fc^2^ calculated for reflections grouped in relative structure factor Fc/Fcmax.
(JPG)

Table S1 **The observed and calculated d-spacings used for unit cell determination.**
(DOCX)

Dataset S1 **The Crystallographic Information File (cif) of dihydrate β-chitin of X-ray and neutron.**
(CIF)
