# Supplementary figures and images for: Water in Crystalline Fibers of Dihydrate β-Chitin Results in Unexpected Absence of Intramolecular Hydrogen Bonding
Source: PLoS One. 2012 Jun 19;7(6):e39376. doi: 10.1371/journal.pone.0039376 (PMC3378566; doi:10.1371/journal.pone.0039376)

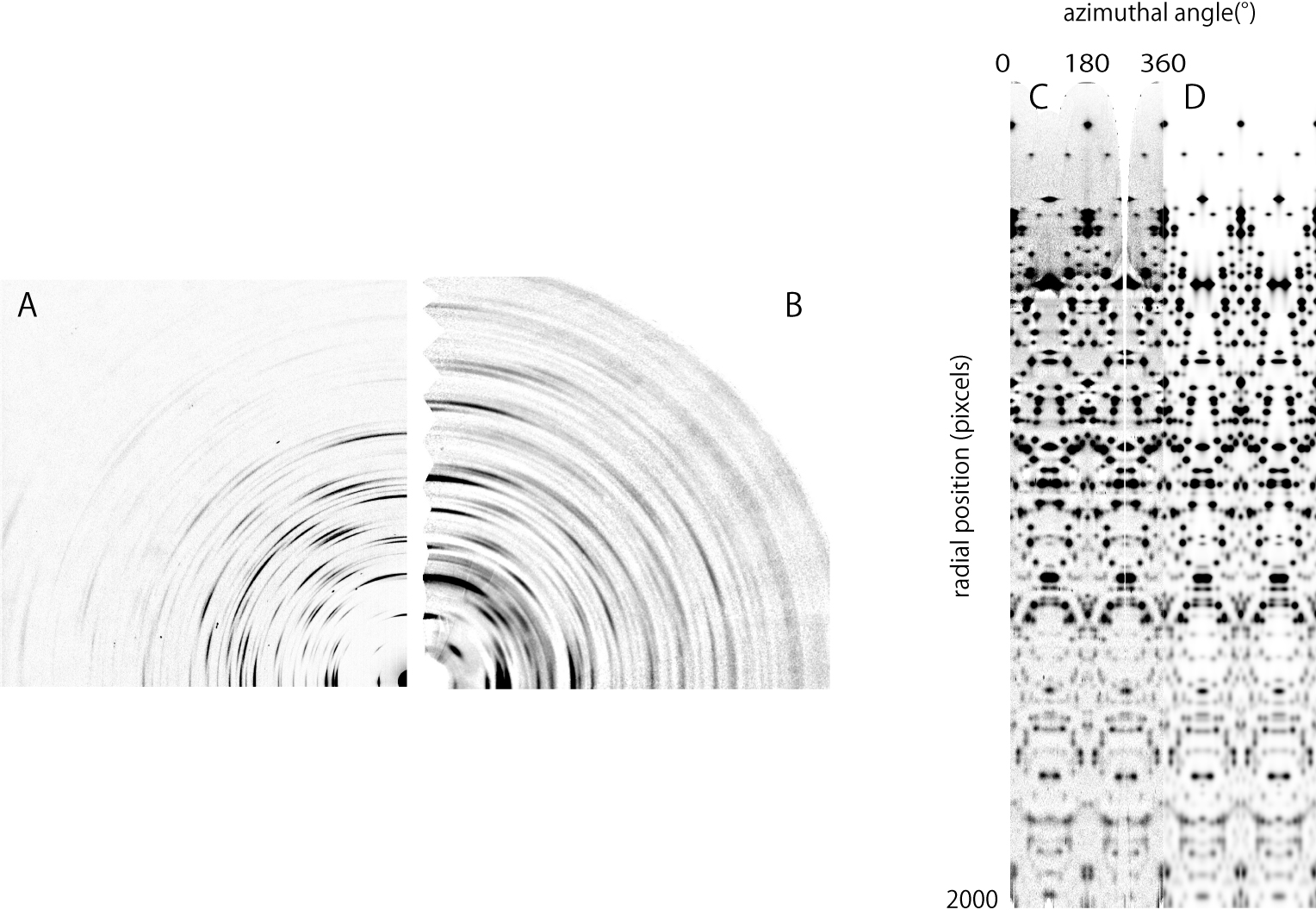

Supplement: Figure S1 — Diffraction data of dihydrate β-chitin X-ray (A) and neutron (B) fiber diffraction data. The fiber direction (meridian) is vertical and the equator is horizontal. Background scattering was subtracted using a rolling-ball algorithm for the purpose of improved visual presentation. X-ray fiber diffraction diagrams transformed into Polar coordinates. (C): observed (D): fitted. The origin of azimuthal angle corresponds to horizontal left of fiber pattern in (A). (JPG) [file pone.0039376.s001.jpg]

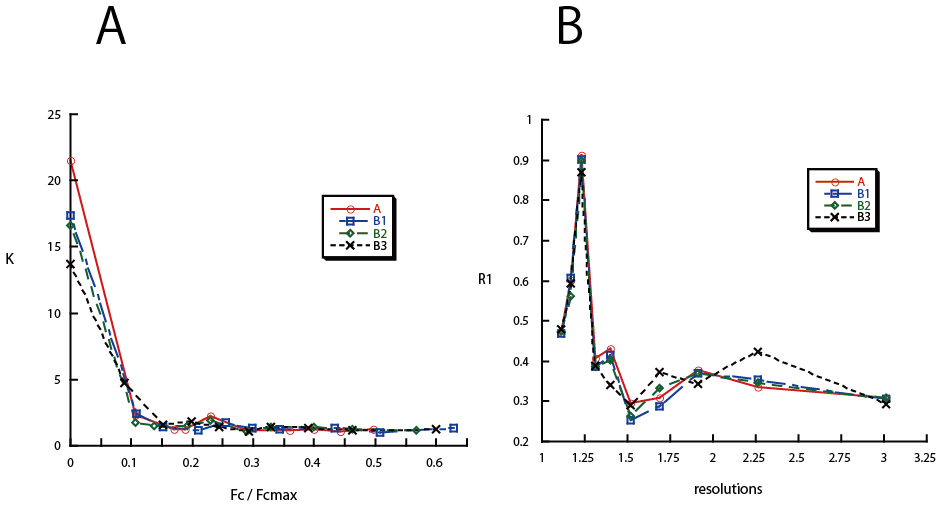

Supplement: Figure S2 — Data selection from R1 and K. (A) R1 values calculated for reflections grouped in resolution shell. (B) K, the mean of Fo2 over the mean of Fc2 calculated for reflections grouped in relative structure factor Fc/Fcmax. (JPG) [file pone.0039376.s002.jpg]
