# Supplementary material for: Water in Crystalline Fibers of Dihydrate β-Chitin Results in Unexpected Absence of Intramolecular Hydrogen Bonding
Source: PLoS One. 2012 Jun 19;7(6):e39376. doi: 10.1371/journal.pone.0039376 (PMC3378566; doi:10.1371/journal.pone.0039376)
Supplement: Table S1 — The observed and calculated d-spacings used for unit cell determination. (DOCX) [file pone.0039376.s003.docx]

| h | k | l | dobs | dcal |
| --- | --- | --- | --- | --- |
| 0 | 1 | 0 | 11.047 | 11.050 |
| 1 | 0 | 0 | 4.771 | 4.788 |
| -1 | 1 | 0 | 4.577 | 4.587 |
| 1 | 1 | 0 | 4.223 | 4.222 |
| -1 | 2 | 0 | 3.852 | 3.841 |
| 1 | 2 | 0 | 3.446 | 3.431 |
| -1 | 3 | 0 | 3.093 | 3.094 |
| 0 | 5 | 0 | 2.220 | 2.210 |
| 0 | 1 | 1 | 7.561 | 7.582 |
| 1 | 0 | 1 | 4.343 | 4.351 |
| -1 | 1 | 1 | 4.211 | 4.199 |
| 1 | 1 | 1 | 3.916 | 3.913 |
| -1 | 2 | 1 | 3.609 | 3.604 |
| 1 | 2 | 1 | 3.268 | 3.259 |
| -1 | 3 | 1 | 2.969 | 2.966 |
| 0 | 0 | 2 | 5.217 | 5.211 |
| 0 | 1 | 2 | 4.702 | 4.713 |
| 0 | 2 | 2 | 3.788 | 3.791 |
| 1 | 0 | 2 | 3.519 | 3.526 |
| -1 | 1 | 2 | 3.447 | 3.443 |
| -1 | 2 | 2 | 3.098 | 3.092 |
| 0 | 3 | 2 | 3.017 | 3.008 |
| 1 | 2 | 2 | 2.874 | 2.866 |
| 0 | 1 | 3 | 3.317 | 3.314 |
| 0 | 2 | 3 | 2.944 | 2.941 |
| -1 | 1 | 3 | 2.773 | 2.770 |
| 1 | 1 | 3 | 2.688 | 2.683 |
| 0 | 3 | 3 | 2.535 | 2.527 |
| -1 | 3 | 3 | 2.312 | 2.311 |
| -1 | 4 | 3 | 2.040 | 2.040 |
| 0 | 0 | 4 | 2.607 | 2.606 |
| 0 | 1 | 4 | 2.537 | 2.536 |
| 0 | 2 | 4 | 2.360 | 2.357 |
| 1 | 0 | 4 | 2.291 | 2.289 |
| 1 | 1 | 4 | 2.221 | 2.217 |
| -1 | 2 | 4 | 2.163 | 2.156 |
| 1 | 2 | 4 | 2.082 | 2.075 |
| -1 | 3 | 4 | 1.996 | 1.993 |
